# Vitamin D Deficiency in Chronic Kidney Disease: Recent Evidence and Controversies

**DOI:** 10.3390/ijerph15081773

**Published:** 2018-08-17

**Authors:** Pedro Henrique Franca Gois, Martin Wolley, Dwarakanathan Ranganathan, Antonio Carlos Seguro

**Affiliations:** 1 Kidney Health Service, Royal Brisbane and Women’s Hospital, Herston QLD 4029, Australia; martin.wolley@health.qld.gov.au (M.W.); dwarakanathan.ranganathan@health.qld.gov.au (D.R.); 2 Medical School, University of Queensland, Herston QLD 4029, Australia; 3 Laboratory of Medical Research-LIM12, Nephrology Department, University of São Paulo School of Medicine, São Paulo, CEP 01246-903, Brazil; trulu@usp.br

**Keywords:** Vitamin D, Vitamin D deficiency, chronic kidney disease, proteinuria

## Abstract

Vitamin D (VD) is a pro-hormone essential for life in higher animals. It is present in few types of foods and is produced endogenously in the skin by a photochemical reaction. The final step of VD activation occurs in the kidneys involving a second hydroxylation reaction to generate the biologically active metabolite 1,25(OH)_2_-VD. Extrarenal 1α-hydroxylation has also been described to have an important role in autocrine and paracrine signaling. Vitamin D deficiency (VDD) has been in the spotlight as a major public healthcare issue with an estimated prevalence of more than a billion people worldwide. Among individuals with chronic kidney disease (CKD), VDD prevalence has been reported to be as high as 80%. Classically, VD plays a pivotal role in calcium and phosphorus homeostasis. Nevertheless, there is a growing body of evidence supporting the importance of VD in many vital non-skeletal biological processes such as endothelial function, renin-angiotensin-aldosterone system modulation, redox balance and innate and adaptive immunity. In individuals with CKD, VDD has been associated with albuminuria, faster progression of kidney disease and increased all-cause mortality. Recent guidelines support VD supplementation in CKD based on extrapolation from cohorts conducted in the general population. In this review, we discuss new insights on the multifactorial pathophysiology of VDD in CKD as well as how it may negatively modulate different organs and systems. We also critically review the latest evidence and controversies of VD monitoring and supplementation in CKD patients.

## 1. Introduction

Vitamin D (VD) is a pro-hormone essential for life in higher animals. It is present in few types of foods and is produced endogenously in the skin by a photochemical reaction [1]. There are two major forms of VD, ergocalciferol (VD_2_) and cholecalciferol (VD_3_), both sharing similar metabolic pathways [2]. VD_2_ is most commonly found in vegetable sources and in “fortified” foods [1,2,3]. VD_3_ can be found in animal-based foods but is mainly synthesized in the skin by a photolytic conversion of cutaneous 7-dehydroxycholesterol by UV sunlight to form previtamin D_3_ and subsequently VD_3_ [4,5].

Regardless of its source, VD_2_ and VD_3_ are transported by a VD-binding protein (VDBP) in the liver where they undergo hydroxylation at the carbon 25 position by 25-hydroxylase (also known as CYP2R1) to become 25-hydroxyvitamin D [25(OH)-VD] [6]. This compound is the main circulating form of VD and its plasma levels are routinely measured as a marker of VD status [2]. Although 25(OH)-VD is considered the precursor of the active form 1,25(OH)_2_-VD, it can also bind to vitamin D receptor (VDR), generating biological responses [7].

**Figure 1:**
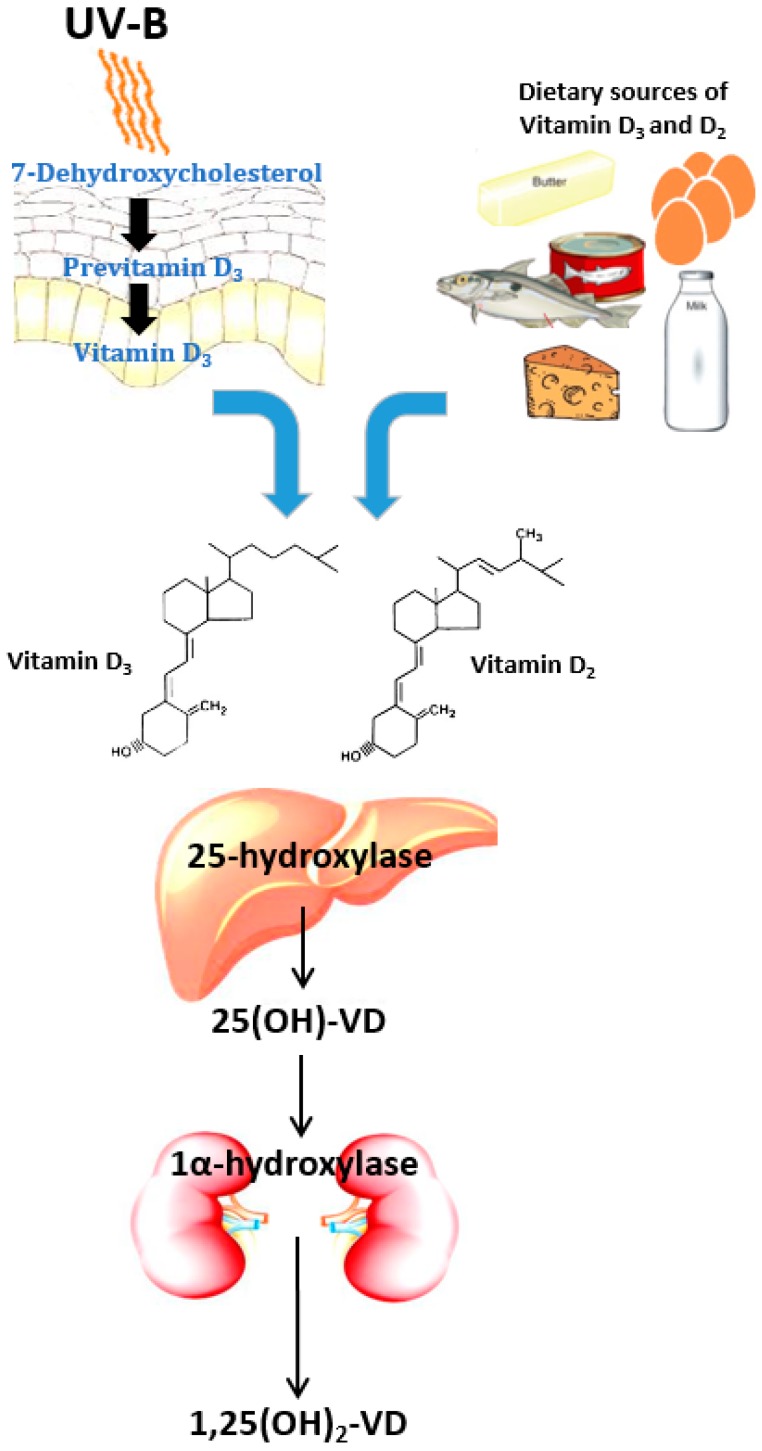
Vitamin D activation and metabolism. Adapted from Gois et al [3].

The final step of VD activation involves a second hydroxylation in which the enzyme 1α-hydroxylase (also known as CYP27B1) converts 25(OH)-VD into 1,25(OH)_2_-VD (Figure 1) [8]. Under physiological conditions, 1,25(OH)_2_-VD is mainly synthesized in the kidneys but in specific conditions, such as pregnancy, chronic renal failure, rheumatoid arthritis and granulomatous diseases, other cell types can also contribute to its circulating levels [6]. Moreover, there is an increasing body of evidence about the pivotal role of extra-renal 1α-hydroxylation for autocrine and paracrine signaling [9,10,11]. Numerous studies have shown 1-α-hydroxylase activity in many tissues including placenta/decidua, pancreas, colon, vasculature, breast and ovary where it may contribute to tissue function, cell proliferation and immunoregulation [9]. Therefore, the importance of VD in many biological processes transcends calcium and phosphate homeostasis.

There is no absolute consensus about the definition of VD sufficiency. According to many experts, serum 25(OH)-VD level should be equal or greater than 75 nmol/L (30 ng/mL) [12]. VD insufficiency (VDI) is defined as a serum 25(OH)-VD level between 50 and 74 nmol/L (20–29 ng/mL), whereas VD deficiency (VDD) is recognized as 25(OH)-VD levels of less than 50 nmol/L (20 ng/mL) [12]. Despite of these definitions, many prevalence studies have employed different cut-offs for VDD, thus causing uncertainty about the magnitude of the problem [13,14,15]. 

On the other hand, the upper normal limit of 25(OH)-VD has been a matter of discussion. Excessive sun exposure has never been reported as a cause of VD intoxication [12]. The highest VD level obtained by sunlight exposure was 225 nmol/L reported in a farmer in Puerto Rico [16], whilst individuals exposed to artificial UVB source showed increased VD level as high as 273.6 nmol/L [17]. VD intoxication can be defined as 25(OH)-VD > 150 ng/mL in combination with hypercalcemia, hypercalciuria and frequently hyperphosphatemia [12]. In fact, 25(OH)-VD levels above 125–150 nmol/L should be avoided, as they might be associated with increased risk of intoxication [18].

Although the mechanisms are not fully elucidated, VD may impact on vascular calcification. Smooth muscle calcification is a well-known complication of the CKD-associated bone mineral disease [19]. Animal and human studies have reported a biphasic curve of VD on vascular calcification with either hypervitaminosis D or VDD potentially correlating with detrimental vascular consequences [20,21].

In individuals with CKD, VDD is highly prevalent and has been associated with albuminuria, faster progression of kidney disease and increased all-cause mortality [22,23,24]. Recent guidelines support 25(OH)-VD supplementations in CKD based on extrapolation from cohorts drawn from the general population [25,26,27,28]. In this review, we discuss new insights on the multifactorial pathophysiology of VDD in CKD, as well as how it may negatively modulate different organs and systems. We also critically review the latest evidence and controversies of 25(OH)-VD monitoring and supplementation in CKD patients.

## 2. VD Deficiency in CKD: Prevalence and Contributing Factors

In the general population, VDD is a well-recognized public health problem worldwide with prevalence ranging from 20% and 100% [3,29,30]. Among the most vulnerable to VDD are the elderly, people living in higher latitudes, people with darker skin, obese individuals and patients with CKD [31].

Several studies have demonstrated that individuals with CKD are at high risk of VDD [32,33,34,35]. Gonzalez et al. reported that 97% of the patients on hemodialysis presented inadequate levels of 25(OH)-VD [33]. In a cross-sectional analysis of a cohort study including 1056 United States dialysis units, Bhan et al. showed that 79% and 57% out of 908 individuals on chronic hemodialysis (HD) had 25(OH)-VD levels of <30 and <20 ng/mL, respectively [36]. Hypoalbuminemia, black color and dialysis initiation during the winter are strong predictors of VDD, whereas VDD was universal in patients presenting with all these three predictors [36]. Furthermore, the prevalence of VDD among patients with stage 3 and stage 4 CKD (not yet on dialysis) was studied in a multi-center cohort from 12 geographically diverse regions of the United States [37]. Strikingly, the investigators found that only 29% and 17% of patients respectively with stage 3 and stage 4 CKD had sufficient 25(OH)-VD levels [37]. 

Although 25(OH)-VD levels start to decrease in individuals with CKD stage 2, inadequate levels can be found in all stages of CKD [33,37,38,39]. Many factors have been implicated in the high prevalence of VDD among CKD patients. 

Patients with CKD, especially on HD, are likely to have less sunlight exposure [38,40]. Del Valle et al. showed that 84% of the HD patients with VDD had inadequate sunlight exposure [40]. Uremia may also blunt the response of plasma VD to UVB irradiation [41]. Chronic HD patients exhibited a lower VD response than normal individuals when exposed to a physiologically equivalent dose of UVB [41]. Furthermore, hyperpigmentation, one of the most common cutaneous manifestations in patients undergoing HD, may play an additional role in the impaired endogenous VD synthesis [38,42].

Nutritional factors may also contribute to suboptimal 25(OH)-VD status in CKD. Patients with CKD frequently have low food intake due to numerous reasons such as reduced appetite, uremic-related gastrointestinal symptoms and dietary restrictions, i.e., low protein (especially in those on conservative management) and low phosphate diets [43,44,45]. Uremia might be associated with impaired gastrointestinal absorption of VD. Vaziri et al. showed using an in vivo perfusion technique that uremic rats had a significantly lower rate of jejunal absorption of labeled VD_3_ compared to control animals [46]. Nevertheless, the authors did not provide any evidence of the potential mechanisms involved in the uremic impairment of VD gastrointestinal absorption and these results are yet to be translated to humans. 

**Figure 2:**
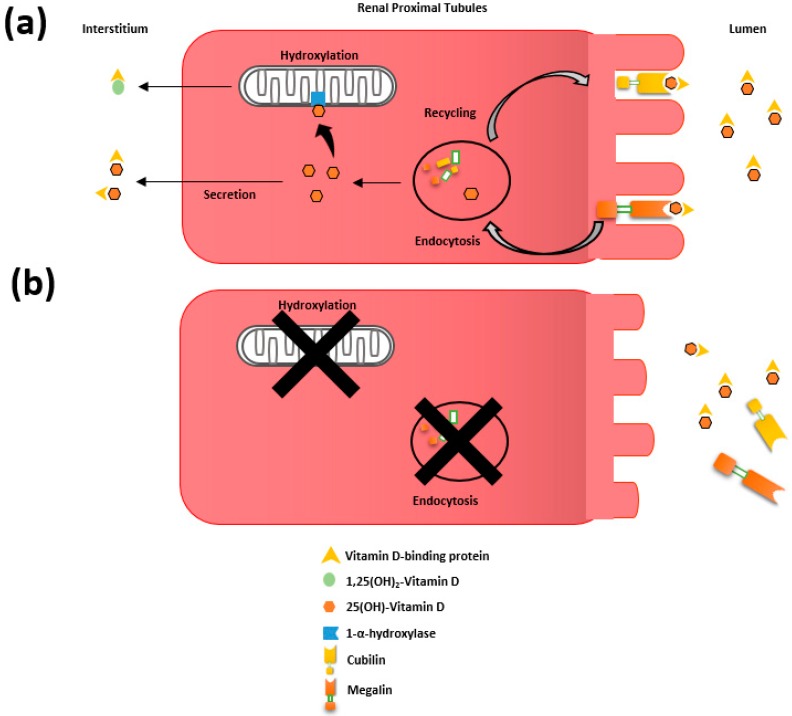
Representation of the tandem function of megalin and cubilin in renal uptake of 25(OH)-VD. (**a**) Filtered complexes of vitamin D binding protein (VDBP) and 25(OH)-VD are endocytosed by the proximal tubular epithelium via an endocytic receptor-mediated pathway recognizing VDBP. The VDBP is degraded in the lysosomes releasing 25(OH)-VD which is either secreted or hydroxylated in the mitochondria to 1,25(OH)_2_-VD. Both 25(OH)-VD and 1,25(OH)_2_-VD reenter the circulation bound to VDBP. (**b**) Postulated megalin and cubilin shedding in chronic kidney disease (CKD) perpetuating vitamin D deficiency (VDD) with subsequent lower 25(OH)-VD reuptake and intracrine 1,25(OH)_2_-VD production in the renal proximal tubules.

Proteinuria has also been described as a contributing factor in the pathogenesis of VDD [2,47]. The 58 kDa VDBP is an alpha globulin that carries more than 85% of the circulating 25(OH)-VD. Complexes of VDBP and 25(OH)-VD are filtered in the glomerulus, allowing transport to the proximal tubule, where a receptor-mediated reabsorption occurs at the level of the brush border involving megalin and cubilin (Figure 2a) [48,49]. Patients with proteinuria usually present with increased urinary excretion of VDBP but might also show impaired megalin and cubilin mediated protein reuptake in the proximal tubules [50,51]. Leheste et al. showed that inactivation of the megalin gene in mice lead to increased urinary excretion of VDBP, VDD, hypocalcemia and osteomalacia [52]. In humans, increased urinary excretion of megalin and cubilin have been reported in diabetes and IgA nephropathy [49,51,53]. Megalin and cubilin shedding therefore might contribute to VDD in the setting of CKD and proteinuria (Figure 2b).

Serum levels of 25(OH)-VD were found to decline progressively with time in patients on peritoneal dialysis (PD) [54]. Some authors reported lower levels of 25(OH)-VD in PD patients compared to those on HD [55,56]. Gokal et al. reported a mean level of 2 nmol/L of 25(OH)-VD in the PD effluent [54]. VDBP has been also detected in peritoneal dialysate [57,58]. Therefore, patients on PD are at particularly high risk for VDD given the increased loss of both 25(OH)-VD and VDBP through the peritoneal effluent [57,58,59].

Diabetes mellitus is one of the most common causes of CKD worldwide. VDD and diabetes share a major attribute: both are pandemic [60]. Lower levels of 25(OH)-VD correlate with insulin resistance and lower β-cell function, whereas 25(OH)-VD supplementation might be associated with lower incidence of type 1 diabetes in children [60,61]. On the other hand, body mass index is inversely correlated with 25(OH)-VD levels, therefore VDD and obesity may be concomitant risk factors for type 2 diabetes [62]. Furthermore, lower 25(OH)-VD levels may predict all-cause mortality in type 1 and type 2 diabetes [63,64]. Altogether, there seems to be an overlapping relationship of risk factors involving VDD, diabetes and CKD. 

## 3. VD: Non-Classical Effects

**Figure 3:**
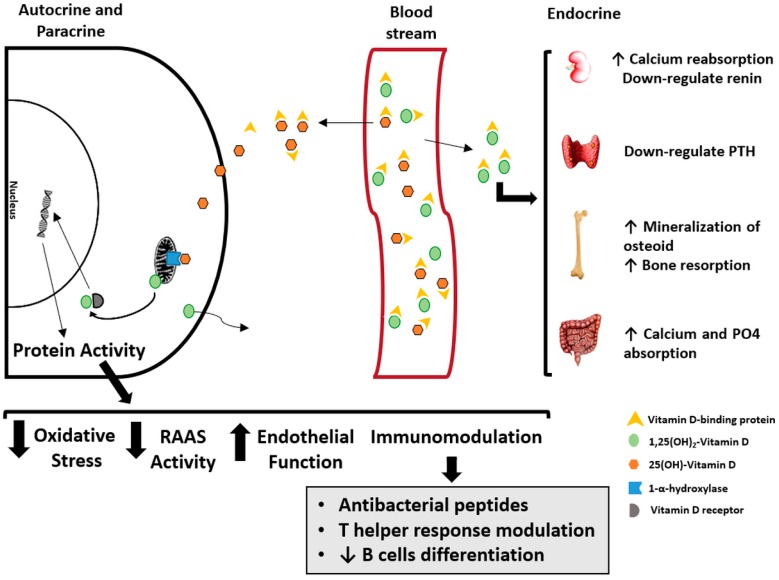
Schematic model of the classical and non-classical effects of vitamin D. The compounds 25(OH)-VD and 1,25(OH)_2_-VD circulate mainly bound to the vitamin D binding protein (VDBP). The endocrine effects of 1,25(OH)_2_-VD are represented on the right. Different types of cells can present the machinery for 25(OH)-VD activation (left). In an autocrine and paracrine fashion, 1,25(OH)_2_-VD regulates the transcription of pivotal proteins involved in several biological processes (left). RAAS = renin-angiotensin-aldosterone system. PTH = parathyroid hormone.

There is a growing body of evidence supporting the importance of VD in many vital non-skeletal biological processes, such as endothelial function, renin-angiotensin-aldosterone system regulation, redox balance and innate and adaptive immunity (Figure 3). These are known as the non-classical effects of VD.

### 3.1. VD and Endothelial Function 

A number of studies have described an association between low 25(OH)-VD levels and endothelial dysfunction [65,66,67,68]. Carrara et al. prospectively compared 33 patients with essential hypertension and normal 25(OH)-VD levels to 33 patients with essential hypertension and VDD who underwent eight weeks of VD supplementation. The VDD subgroup had a significant increase in flow-mediated dilation (FMD) of the brachial artery, an important research tool for assessment of endothelial function in vivo [66]. However, in a systematic review, only two out of ten randomized clinical trials (RCTs) reported that VD supplementation ameliorated FMD [68].

### 3.2. VD and the Renin-Angiotensin-Aldosterone System

Vitamin D has also been implicated as an agent which can modulate the renin-angiotensin-aldosterone system (RAAS), and therefore which may influence blood pressure and cardiovascular disease. Evidence for this interaction comes from animal models, molecular studies and clinical data. 

In one animal study, vitamin D receptor null mice were generated and demonstrated upregulation of renin and angiotensin II, as well as significant hypertension, increased water intake and increased left ventricular mass compared to wild type animals [69]. Further supporting these findings, 1,25(OH)_2_-VD supplementation suppressed renin production in a separate group of wild type animals. Other studies have also demonstrated that paricalcitol supplementation decreases renin and renin receptor expression in animal models of CKD [70]. The mechanism by which this interaction occurs is not yet completely elucidated, but the VDR appears to be able to interact directly with elements of the intracellular complex, which promotes pro-renin transcription when in a 1,25(OH)_2_-VD ligand-bound form [71]. The interaction has the effect of suppressing renin gene expression, thus suggesting a plausible mechanism.

Whilst these data suggest a role for VD in RAAS regulation, human data linking VDD with hypertension as an end-point of RAAS activation have been mixed. Seasonal and regional blood pressure trends suggest a relationship between UV exposure and hypertension, and cross-sectional studies have demonstrated that VD levels correlate with hypertension prevalence, supporting a VD-RAAS link [72]. However, the largest meta-analysis summarized 46 prospective trials and suggested no effects of 25(OH)-VD supplementation on blood pressure [73]. This does not completely exclude a role for VD in modulation of the RAAS but suggests that the effect may be small and possibly subclinical. Concerns about heterogeneous methods of 25(OH)-VD supplementation, variable achieved 25(OH)-VD levels and variable levels of baseline VDD in the existing trials have caused some uncertainty however, and several trials are ongoing.

### 3.3. VD and Redox Balance

Low levels of 25(OH)-VD have been associated with increased markers of oxidative stress. In different experimental models, VD deficient animals showed increased thiobarbituric acid reactive substances (TBARS) and decreased glutathione (GSH) levels, a biomarker of oxidative stress and a major endogenous antioxidant, respectively [74,75,76]. Furthermore, human observational studies have shown an inverse relationship between 25(OH)-VD levels and reactive oxygen species [65,77]. Despite these promising results, further clinical studies need to be undertaken to verify whether there is a beneficial effect of VD supplementation on redox balance in subjects with low 25(OH)-VD levels.

### 3.4. VD and the Immune System

Previous in vitro studies highlighted monocytes and macrophages as one of the first non-renal cells with the ability not only to synthesize 1,25(OH)_2_-VD, but also to upregulate the expression of 1α-hydroxylase [9,10]. Once in the monocytes, 25(OH)-VD is converted to active 1,25(OH)_2_-VD by mitochondrial 1-α-hydroxylase and binds to cytoplasmic VDR, thereby acting as a transcription factor for antibacterial peptides such as cathelicidin and beta-defensin 4A. [3,78,79]. More recently, the machinery for VD activation was also observed in other antigen-presenting cells such as dendritic cells [10,80].

It has been shown that 1,25(OH)_2_-VD may also have an anti-inflammatory effect in human T cells [81], as 1,25(OH)_2_-VD has been reported to reduce the expression of the nuclear factor κB (NFκB). In addition, 1,25(OH)_2_-VD may promote a shift in the T helper (Th) cell response from Th1 to Th2, subsequently reducing Th1-mediated tissue damage and increasing the production of Th2 immunomodulatory cytokines [82,83]. Moreover, some studies have reported expression of VDR, 1α-hydroxylase and 24-hydroxylase in human B cells [83,84]. There is also evidence that 1,25(OH)_2_-VD may inhibit the differentiation of B cells into plasma cells, thus modulating the production of antibodies [82,83].

## 4. VD and CKD: Human Studies

### 4.1. Bone Mineral Disease and Muscle Health

The inverse correlation between 25(OH)-VD levels and parathyroid hormone (PTH) has been demonstrated across virtually all stages of CKD [23,85,86]. The prevalence of secondary hyperparathyroidism almost doubled when non-dialysis patients presented with 25(OH)-VD ≤ 20 ng/mL compared to those with levels > 20 ng/mL [32]. In addition, PTH levels seem to plateau when 25(OH)-VD is greater than 30 ng/mL [32].

A systematic review with meta-analysis of observational and randomized studies showed a significant decline in PTH levels with 25(OH)-VD supplementation [87]. Similar results were obtained when patients with CKD received active VD analogs [88,89]. Indeed, treatment with either 25(OH)-VD or active VD analogs induced similar responses on PTH in patients with CKD stage 3–4 and hyperparathyroidism [90]. These results suggest a potential additive effect of 25(OH)-VD and active VD analogs on renal hyperparathyroidism [90]. 

Low 25(OH)-VD has been linked with increased bone turnover and decreased bone mineral density (BMD) in patients with CKD. In a cohort study including 1,026 non-dialysis patients across all CKD stages, Ureña-Torres et al. showed that 25(OH)-VD ≤ 15 ng/mL was associated with high serum bone-specific alkaline phosphatase (BALP) and C-terminal cross-linked collagen type I telopeptides (CTX), both circulating bone remodeling biomarkers [91]. Similar results were reported by Yadav et al. who found that 25(OH)-VD supplementation reduced PTH, BAP and CTX in a randomized, double blind, placebo-controlled trial including 117 patients with CKD stage 3–4 [92]. Levels of 25(OH)-VD ≤ 20 ng/mL were also associated with lower BMD at the femur neck and total hip in individuals with CKD stages 3–4 in a Korean populational cohort [93]. 

There is evidence that 25(OH)-VD may hold a direct and independent role on bone formation and mineralization. Coen et al. retrospectively analyzed bone histomorphometry and histodynamic for different levels of 25(OH)-VD in a cohort of 104 patients on hemodialysis for more than 12 months [94]. The investigators found that 25(OH)-VD < 20 ng/mL was associated with relatively low bone turnover, whereas histologic evidence of a mineralization defect was only found when VDD was accompanied by elevated PTH [94]. Moreover, patients on HD have twice the risk of symptomatic bone fracture compared to renal transplant patients [95].

There has been a growing interest in the potential role of VD modulating metabolic pathways implicated in muscle function [96]. Osteomalacia is a well-known cause of proximal myopathy predominantly with atrophy of type II muscle fibers [97]. Sarcopenia is highly prevalent among CKD patients and is associated with increased morbidity and mortality [98,99]. Nevertheless, the role of lower 25(OH)-VD levels in the pathogenesis of CKD-related sarcopenia remains unclear. Souza et al. did not find significant difference in 25(OH)-VD levels in patients with CKD and sarcopenia compared to those non-sarcopenic [100]. In patients with end stage renal failure, low 25(OH)-VD has also been associated with muscle weakness and risk of falls, but the evidence to support these associations is limited to small observational studies [101,102].

Overall, despite the potential benefits of 25(OH)-VD on biochemical markers of mineral metabolism, there is insufficient RCT data available showing unequivocal benefits of supplementation on muscle strength, risk of falls and prevention of fractures in individuals with CKD.

### 4.2. Albuminuria

Several recent observational studies have highlighted the importance of 25(OH)-VD in areas outside of traditional bone and mineral metabolism. A cross-sectional analysis of the Third National Health and Nutrition Examination Survey (NHANES III) revealed a progressively higher prevalence of albuminuria with decreasing 25(OH)-VD levels in a representative sample of the US population [22]. These results supported the findings of previous studies enrolling diabetic patients in Italy and Japan [103,104]. In Australia, Damasiewicz et al. conducted a prospective study including 6180 adults with normal renal function at baseline from the Australian Diabetes, Obesity and Lifestyle (AusDiab) study [105]. This large population-based cohort with two follow up phases (at baseline and five-year) showed that individuals with 25(OH)-VD levels < 15 ng/mL had increased incidence of albuminuria, defined as spot urine albumin-creatinine ratio ≥ 2.5 mg/mmol for men and ≥ 3.5 mg/mmol for women [105]. There was a consensus among these studies around the stepwise increase in the prevalence of albuminuria with decreasing 25(OH)-VD levels, however, a clear cutoff point could not be determined.

VD has been shown to suppress the transcription of renin, inhibiting the RAAS and ultimately leading to a reduction in proteinuria through hemodynamic and non-hemodynamic pathways [69,106,107,108]. VD may also modulate oxidative stress and inflammation, reducing fibroblast activation and interstitial inflammation [74,75,77,109] Moreover, CKD progression and lower expression of megalin have been associated with lower 25(OH)-VD reuptake and therefore reducing intracrine 1,25(OH)_2_-VD production in the renal proximal tubules (Figure 2b) [50,51,110]. On the other hand, increasing levels of proteinuria may perpetuate VDD. Altogether, there seems to be a synergistic interplay between VDD and CKD leading to a vicious cycle for progressive deterioration of renal function.

Molina et al. published a well-designed single-centre, controlled trial enrolling individuals with CKD stage 3–4 and persistent albuminuria. Patients were assigned to receive 666 IU of VD_3_ daily, regardless of the 25(OH)-VD levels, when the PTH was above the expected range for the stage of CKD. Fifty patients were allocated to the intervention group and 51 patients received no intervention. Despite of the small dose of VD_3_, the authors found a 53% reduction in the urine albumin:creatinine ratio after six months of VD_3_ treatment [111]. Similarly, Kim et al. reported an anti-proteinuric effect of VD_3_ in patients with concomitant diabetes, CKD stage 2–4 and low 25(OH)-VD in a small observational study [112]. Nevertheless, no RCT assessing the effects of 25(OH)-VD supplementation on albuminuria has been published thus far. We identified one ongoing study (ClinicalTrials identifier NCT01029002) enrolling 75 patients with CKD stage 3–4 to receive either VD_2_ or placebo for the primary outcome change in the proteinuria status.

### 4.3. CKD Progression and Mortality

Recently, many observational studies have examined the association between lower 25(OH)-VD, CKD progression and mortality. Ravani et al. followed up 168 consecutive new referrals to a CKD clinic over a period of six years. CKD stages ranged from 2–5 pre-dialysis, and most patients had stage 3 and stage 4 CKD. The levels of 25(OH)-VD predicted progression to dialysis and death in crude analysis and in multiple regression models [23]. Similarly, Barreto et al. conducted a prospective study including 140 CKD patients from stage 2–5. The authors aimed to investigate the association between VD levels, vascular calcification, endothelial function and mortality. Although there was an association between 25(OH)-VD levels and mortality, the investigators did not find significant correlation between 25(OH)-VD, aortic calcification and pulse wave velocity [39]. Moreover, Wolf et al. performed a cross-sectional analysis of 825 consecutive incident hemodialysis patients across 569 hemodialysis centres in 37 states in the USA [24]. Patients who died within 90 days of initiating dialysis were compared with those who survived for at least 90 days. Individuals presenting with 25(OH)-VD < 10 ng/mL were at significantly increased risk of all-cause and cardiovascular mortality compared to subjects with 25(OH)-VD > 30 ng/mL, whilst subjects with 25(OH)-VD levels 10–30 ng/mL showed mixed results after multivariate adjustments [24].

Altogether, despite the observational studies highlighting the role of VDD as a potential risk factor for progression of CKD and mortality, we did not identify any RCT aiming to verify whether there is a beneficial effect of 25(OH)-VD supplementation on these outcomes.

## 5. VD and CKD: Current Guidelines

Both the Kidney Disease Outcomes Quality Initiative (KDOQI) and Kidney Disease Improving Global Outcomes (KDIGO) experts recommend checking and supplementing low serum 25(OH)-VD levels in CKD and dialysis patients [25,26]. In the most recent update of the KDIGO guidelines on bone mineral disorder, it is suggested based on low quality evidence that patients with CKD stage 1–5 have 25(OH)-VD levels measured, and repeated testing should be individualized according to baseline values and interventions [25]. Nevertheless, there was no clear suggestion on how frequently 25(OH)-VD levels should be reviewed [25]. 

With respect to the recommended dietary allowance of VD in the general population, the Institute of Medicine from the US and Canada recommended that adults up to the age of 70 years require 600 IU/d of VD, whereas adults 71 years and older require 800 IU/d [113]. These recommendations cover the needs of >97.5% of the population and assume minimal or no sun exposure, thus providing further safety for individuals with lower endogenous synthesis of VD [113]. 

Current guidelines suggest that patients with CKD stage 1–5 and VDD or VDI should receive supplementation using the same strategies as recommended for the general population [25,26,114]. However, even for the general population, the optimal dosage of supplementation varies among the main guidelines. The KDOQI suggests 1000–2000 IU/d of VD_3_ for VD repletion, but acknowledges that patients with CKD may require a more aggressive therapeutic plan [26]. The National Institute for Clinical Excellence (NICE) in the UK suggests that people aged ≥ 65 years who are not exposed to much sun should take 400 IU of VD_3_ daily, nevertheless, this guideline did not address VD supplementation in individuals with VDD or VDI [27]. In Australia and New Zealand, the Kidney Health Australia-Caring for Australasians with Renal Impairment (KHA-CARI) does not suggest any specific dosage for VD repletion [114].

Another matter of debate is around which form of VD should be used. VD_2_ and VD_3_ undergo identical hydroxylation processes and in theory are equally used by the body to generate 1,25(OH)_2_-VD [115]. In fact, their chemical structure only differs in the side chains (Figure 1) [116]. Armas et al. compared the potency of a single dose of 50,000 IU VD_2_ and VD_3_ in 30 healthy subjects. Both VD analogues produced similar initial increments in serum 25(OH)-VD, but individuals treated with VD_3_ had a more sustained response with a three-fold difference in the area under the curve on the 28th day [117]. Several theories have been proposed to explain the difference between the two calciferols. VD_3_ might have a higher affinity to both VDR and 25-hydroxylase [118,119]. Other studies have suggested a lesser affinity of VD_2_ for VDBP compared to VD_3_, resulting in higher clearance and subsequently a shorter circulating half-life [120,121,122]. Recently, a meta-analysis including seven heterogeneous studies indicated that regardless of the dosage, frequency or administration (oral or intramuscular), VD_3_ was more effective at raising serum 25(OH)-VD concentrations compared to VD_2_ [123]. Four studies that applied bolus doses also favored VD_3_ over VD_2_, whereas there was no statistical difference between VD_3_ and VD_2_ in the pulled data from studies that used daily supplementation [123]. Although VD_3_ may be more effective than VD_2_, clinicians should ultimately use the presentation commercially available in the context of their clinical practice. For instance, VD_2_ is mostly used in the United States, whilst in other countries, such as Australia and Brazil, VD_3_ is the most common presentation.

## 6. Conclusions

In summary, the studies reviewed here highlight the potential role of VD beyond bone mineral disease in patients with CKD. Currently, the strongest available evidence supports 25(OH)-VD supplementation, aiming to control secondary hyperparathyroidism in CKD patients. Despite the striking observational data showing the association between lower levels of 25(OH)-VD and various deleterious outcomes (such as low bone turnover, risk of falls and factures, albuminuria, progression of CKD and mortality), there is still a lack of RCTs supporting the potential beneficial effects of supplementation. Many questions remain unanswered regarding the dosing, timing of administration and type of VD analogues in patients with CKD. In addition, the current guidelines are subject to criticism for being mainly opinion-based and derived from observational data. However, given the low-cost and high safety profile, patients with CKD might benefit from 25(OH)-VD supplementation in the setting of VDD and VDI. Although doses of up to 4,000 IU of VD_3_ are considered safe for the general population [124], we recommend caution in renal patients, especially in those who are on calcium-containing phosphate binders and/or on active VD analogues.
